# Hospital Meetings

**Published:** 1906-05-12

**Authors:** 


### HOSPITAL MEETINGS.

### BELGRAVE HOSPITAL FOR CHILDREN.

The annual general meeting of this hospital was held at Grosvenor House on May 8, and the chair was taken by Mr. H. C. Good, Vice-Chairman of the Committee of Management. The adoption of the report was moved by the Chairman. He said, as they all knew, a few years ago the hospital had been transferred to the South of London, and one of the drawbacks of South London was that it was so difficult to get people to go down there to take part in the work. The wisdom of transferring the hospital had been amply justified, and it was now doing very well. The Committee felt very grateful to the Council of King Edward's Fund for the special commendation they had given them on account of having moved their hospital, and they had also given them a good contribution towards the expenses of moving. They were now getting into touch with the City companies, and had received the sum of £250 from the Goldsmiths Company. As yet their hospital did not seem to appear in people's wills as much as he would like it to. They had four wants, which were more or less urgently pressing. First, they wanted more guinea subscribers for the purpose of paying the household bills; second, they wanted some charitable testators; third, they wanted to complete the hospital, of which as yet only three-fifths was finished, and it was far more expensive and inconvenient to use a half-completed building; fourth, they wanted a wider circle of friends, and should spread their nets as far as possible. They had raised a large amount of capital, but they had spent it all. They increased their funds each year, but their needs increased more rapidly still. If the hospital was to fulfil its real purpose, and not be cramped, they must have more money for its completion.

Mr. T. A. F. Kingscott seconded the motion, and begged the governors to go down to the hospital and thus get into personal touch with the work, and see for themselves how admirably it was carried on.

Mr. Percy Armytage, Chairman of the House Committee, who also supported the motion, stated that it was not the large subscriptions, but the multitude of small ones that helped on the work of the hospital. They had been most fortunate in donations during the past year, and it was satisfactory to think that they were viewed with favour by the Committee of King Edward's Hospital Fund, who had doubled their grant last year. They were very much indebted to the Ladies' Association who had come to the rescue of the hospital when it was in a very bad plight financially, and had put it in a position to continue its work as before.

Mr. M. S. Duke proposed the re-election of the two treasurers, which was seconded by Lord William Seymour.

Lady Gifford, in proposing the re-election of four retiring members of the Committee, touched upon the subject of infant mortality, which, she affirmed, was a disgrace to England. The motion was adopted, as were various others, and the proceedings terminated with votes of thanks to the Duke of Westminster and the Chairman.

### CHILDHOOD SOCIETY.

The ninth annual meeting of the Childhood Society was held on Tuesday, May 8, at 7 St. James's Square, the chair being taken, in the absence, through illness, of the President, Lord Egerton of Tatton, by Mr. J. W. Palmer, a Vice-President.

The Chairman moved the adoption of the report, and said that, though it was only the ninth, that did not represent the period during which the Society, in various forms, had been at work. Originally the Society had been one for scientific research, and one of their earliest undertakings was to send out cards, on which were obtained a record of the nerve and physical signs of some hundred thousand children. They had always set facts before deductions. Their Society was the practical pioneer of the new science, and he considered it had a great future before it. Great discussions were going on now about education, and there was arising a feeling that it should be looked upon from a different point of view from that of officialdom. They had in the papers read before them, and recorded in their transactions, a remarkable series of new ideas. In the title of the address at their last annual meeting, "Teaching Common Sense in the School of the Future," for instance, they had a subject he was sure would one day bear a great amount of fruit. He thought the Society might in the future have functions they could not at present foresee.

Dr. Shuttleworth seconded the adoption of the report, which he thought constituted a record of an amount of good, honest work of a comprehensive character of which they might be proud. Their income was a small one, but they had laid it out to good purpose. The scientific basis of education had been the leading thought throughout their proceedings. They tried to learn what the material to be educated was by observing the child as one would study any object of natural science. Much had come of their original examination of a hundred thousand children, and teachers were coming to look on the members of their classes as individuals, and that each child had a differential equation. Of course, each child could not be separately taught, but each might be separately considered. One useful piece of work they had been engaged on was, in conjunction with the Child-Study Association, the making of inquiry into the qualifications of teachers. They would not, of course, print anything reflecting on any individual school, but they had come to the conclusion that in many cases there was not an adequate teaching staff. In common with the Child-Study Association, they had been the guests of the Royal Sanitary Institute, and had had a very interesting and instructive winter course. In the summer the business of the committees and investigations went on, and they had organised for them an interesting visit to some institution; and this year they were invited to inspect the reconstructed establishment at Darenth for imbecile children.

The resolution was carried, and the Chairman called on Sir Edward Brabrook to address the meeting.

In the course of the address he said he particularly wished to bring to their notice the forthcoming International Congress on School Hygiene, in which they were specially interested, as having taken part in the first Congress at Nuremburg in 1904. The Council agreed that the next Congress should take place in London, and the date had been fixed for 1907. They were to be honoured by the patronage of the King, and Sir Lauder Brunton had been nominated as President of the Congress. That it was certain to be a most interesting and instructive assembly was shown by the list of subjects for consideration.

The usual vote of thanks closed the meeting.

